# Frequency of *Propionibacterium acnes* Infection in Prostate Glands with Negative Biopsy Results Is an Independent Risk Factor for Prostate Cancer in Patients with Increased Serum PSA Titers

**DOI:** 10.1371/journal.pone.0169984

**Published:** 2017-01-12

**Authors:** Tomoya Kakegawa, Yuan Bae, Takashi Ito, Keisuke Uchida, Masaki Sekine, Yutaka Nakajima, Asuka Furukawa, Yoshimi Suzuki, Jiro Kumagai, Takumi Akashi, Yoshinobu Eishi

**Affiliations:** 1 Department of Human Pathology, Graduate School and Faculty of Medicine, Tokyo Medical and Dental University, Tokyo, Japan; 2 Division of Surgical Pathology, Tokyo Medical and Dental University Hospital, Tokyo, Japan; 3 Department of Pathology, Yokohama City Minato Red Cross Hospital, Yokohama, Japan; University of Ulster, UNITED KINGDOM

## Abstract

**Background:**

*Propionibacterium acnes* has recently been implicated as a cause of chronic prostatitis and this commensal bacterium may be linked to prostate carcinogenesis. The occurrence of intracellular *P*. *acnes* infection in prostate glands and the higher frequency of *P*. *acnes*-positive glands in radical prostatectomy specimens from patients with prostate cancer (PCa) than in those from patients without PCa led us to examine whether the *P*. *acnes*-positive gland frequency can be used to assess the risk for PCa in patients whose first prostate biopsy, performed due to an increased prostate-specific antigen (PSA) titer, was negative.

**Methods:**

We retrospectively collected the first and last prostate biopsy samples from 44 patients that were diagnosed PCa within 4 years after the first negative biopsy and from 36 control patients with no PCa found in repeated biopsy for at least 3 years after the first biopsy. We evaluated *P*. *acnes*-positive gland frequency and *P*. *acnes*-positive macrophage number using enzyme-immunohistochemistry with a *P*. *acnes*-specific monoclonal antibody (PAL antibody).

**Results:**

The frequency of *P*. *acnes*-positive glands was higher in PCa samples than in control samples in both first biopsy samples and in combined first and last biopsy samples (P < 0.001). A frequency greater than the threshold (18.5 and 17.7, respectively) obtained by each receiver operating characteristic curve was an independent risk factor for PCa (P = 0.003 and 0.001, respectively) with odds ratios (14.8 and 13.9, respectively) higher than those of serum PSA titers of patients just before each biopsy (4.6 and 2.3, respectively). The number of *P*. *acnes*-positive macrophages did not differ significantly between PCa and control samples.

**Conclusions:**

These results suggested that the frequency of *P*. *acnes*-positive glands in the first negative prostate biopsy performed due to increased PSA titers can be supportive information for urologists in planning repeated biopsy or follow-up strategies.

## Introduction

Cancer in several organs, including the stomach, liver, and large intestine, has been linked to chronic infection and inflammation. Evidence from epidemiologic, histopathologic, and molecular pathologic studies indicates that chronic inflammation also contributes to prostate cancer [1,2]. *Propionibacterium acnes* is a commensal bacteria that is frequently detected in prostate tissue with prostatitis and prostate cancer (PCa) [3–6]. *P*. *acnes* infection changes cell proliferation, and enables epithelial cells to grow in an anchorage-independent manner, which can lead to cellular transformation [3]. Thus, *P*. *acnes* infection is likely involved in the initiation and/or progression of PCa.

We recently created an anti-*P*. *acnes* monoclonal antibody (PAL antibody) that recognizes an epitope of the lipoteichoic acid that is shared by all strains of phylotype I *P*. *acnes* [7]. This PAL antibody could be used in enzyme immunohistochemistry (IHC) to detect *P*. *acnes* within non-cancerous glandular epithelium and stromal macrophages in formalin-fixed paraffin-embedded (FFPE) prostate samples [7]. Examination of radical prostatectomy specimens from patients with PCa and age-matched control patients with bladder cancer, but without PCa, using the PAL antibody revealed that *P*. *acnes*-positive glands are found more often in PCa specimens than in control specimens. Double labeling of cytoplasmic *P*. *acnes* and nuclear NF-kB expression in prostate tissue sections revealed that NF-kB expression is also more frequent in *P*. *acnes*-infected glands than in glands without *P*. *acnes* infection [7]. These results suggested that latent intraepithelial *P*. *acnes* infection in non-cancerous prostate glands contributes to carcinogenesis in the prostate.

In the present study, we evaluated the implication of the prostate *P*. *acnes* infection status in the risk assessment for patients with negative results from a first prostate needle biopsy performed due to an increased serum PSA titer. For this purpose, we retrospectively collected the first and last prostatic needle biopsy samples from patients with PCa that was diagnosed within 4 years after the first negative biopsy and from control patients with no PCa found in repeated biopsy for at least 3 years after the first negative biopsy. We used enzyme IHC with the PAL antibody to evaluate the number of prostate glands and macrophages that were positive for *P*. *acnes*. With the results from the first biopsy samples and all samples from the first and last biopsy combined, respectively, a receiver operating characteristic (ROC) curve in the final diagnosis of PCa was made, and univariate and multivariate logistic regression analysis were performed to evaluate risk factors for PCa with the frequency of *P*. *acnes*-positive glands, the number of *P*. *acnes*-positive macrophages, the grade of chronic prostatitis of each biopsy sample, and with the serum PSA titer of the patient just before each biopsy.

## Materials and Methods

### Ethics statement

The ethics committee of the Tokyo Medical and Dental University approved the study (Registration No. 1373). The study utilized clinically-obtained and archived FFPE tissue specimens, therefore, the ethics committee waived the requirement for specific informed consent in accordance with Ethical Guidelines for Clinical Studies (amended July 31, 2008) by the Ministry of Health, Labour, and Welfare of Japan.

### Samples

**Fig 1:**
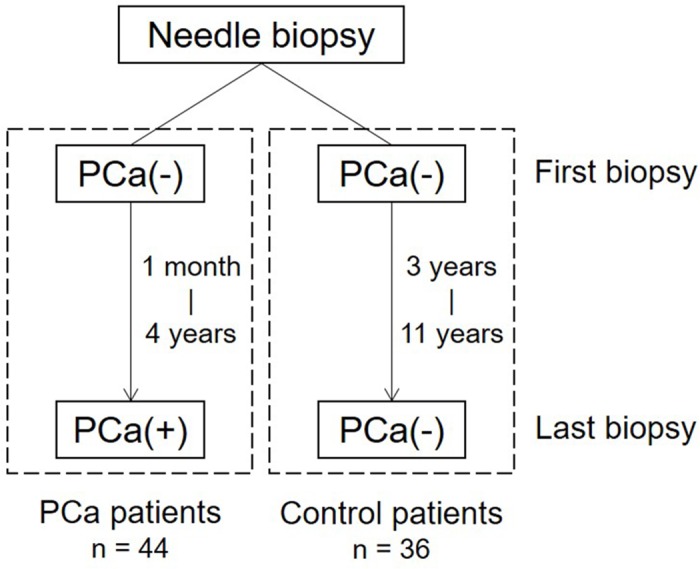
Time line diagram for the biopsy sampling. PCa(-) means negative biopsy where no cancer lesion was found in any cores of the biopsy sample. PCa(+) means positive biopsy where cancer lesion was found in one or more cores of the biopsy sample. Interval between the first and last biopsy varied from 1 month to 4 years in the PCa patients and from 3 years to 11 years in the control patients.

We examined FFPE tissue sections of prostatic needle biopsy samples from 44 patients (age: 52–83 years) with PCa found in repeated biopsy for 1 month or more (up to 4 years) after the first negative biopsy between 1998 and 2014 at the Tokyo Medical and Dental University Hospital (Fig 1). We also examined FFPE tissue sections of prostatic needle biopsy from 36 control patients (age: 40–79 years) with no PCa found in repeated biopsy for 3 years or more (up to 11 years) after the first negative biopsy between 1997 and 2014 at the same hospital. The clinicopathologic profiles of the PCa patients and the control patients are shown in Table 1. All patients were suspected to have PCa based on an increased serum PSA titer (> 4 ng/ml), and had undergone prostate needle biopsy two or three times due to a negative first biopsy. Prostate needle biopsy was performed by the transrectal [8] or transperineal [9] method, or a combination of these methods [10,11]. Samples from the first and last needle biopsy were used for all patients enrolled in the study. The median number of biopsy cores for the first and last biopsy was 14 and 18, respectively, in the patients with PCa and 24 and 18, respectively, in the patients without PCa. Serum PSA titers of patients just before the first and last biopsy were used for all patients enrolled in the study.

**Table 1:** Clinicopathologic profiles of the patients.

Profiles	Value	Profiles	Value
Prostate cancer patients (n = 44)		Control patients (n = 36)	
Age, year, mean ± SD		Age, year, mean ± SD	
First biopsy	66.6 ± 6.8	First biopsy	62.7 ± 7.0
Last biopsy	68.4 ± 6.4	Last biopsy	67.7 ± 6.6
Number of cores, mean ± SD		Number of cores, mean ± SD	
First biopsy	13.5 ± 7.4	First biopsy	19.2 ± 7.6
Last biopsy	19.2 ± 6.0	Last biopsy	19.3 ± 5.3
Sampling method		Sampling method	
First biopsy		First biopsy	
TP+TR	19	TP+TR	22
TP	9	TP	9
TR	14	TR	4
Indefinite	2	Indefinite	1
Last biopsy		Last biopsy	
TP+TR	24	TP+TR	26
TP	17	TP	10
TR	3	TR	0
Interval of each biopsy, year		Interval of each biopsy, year	
< 1	11	3–4	16
1–2	15	4–6	10
2–3	11	6–8	8
3–4	7	≥8	2
Gleason score			
3–5	5		
6	12		
7	12		
8	9		
9	6		
PSA, ng/ml, mean ± SD		PSA, ng/ml, mean ± SD	
First biopsy	15.5 ± 20.8	First biopsy	7.7 ± 3.7
Last biopsy	17.9 ± 17.6	Last biopsy	12.1 ± 7.4

SD: standard deviation, TP: transperineal, TR: transrectal, PSA: prostate-specific antigen

### Immunohistochemistry and histologic evaluation

The IHC procedure with the PAL antibody was performed as previously described [7]. IHC with the PAL antibody detects both phylotype I and II *P*. *acnes* (S1 Fig), although the PAL antibody reacts with only phylotype I *P*. *acnes* when examined by Western blotting with sonicated bacterial lysate. The number of prostate glands with intracellular *P*. *acnes* infection was determined under high-power light microscopy instead of by virtual slides as used in the previous study [7], because of our intention to make this method available to other standard pathologic laboratories. Each gland was considered *P*. *acnes*-positive when we observed PAL antibody-dependent positive signals in the cytoplasm of at least one epithelial cell of the gland. All of the glands included in all cores of tissues obtained by each needle biopsy were determined to be *P*. *acnes*-positive or negative, and the frequency (%) of *P*. *acnes*-positive glands for each biopsy sample was calculated as the total number of *P*. *acnes*-positive glands divided by the total number of glands counted times 100. Cancer glands present in core(s) with cancer lesions in the last biopsy samples from PCa patients were excluded from the counting. All of the prostatic stromal cells with PAL antibody-positive signals in the cytoplasm were counted using a light microscope for all cores of prostate tissues and expressed as the mean number of *P*. *acnes*-positive macrophages per core for each biopsy. Glands and stroma were discriminated using IHC sections counterstained with Mayer’s hematoxylin. We used the criteria of Cohen et al. [5] to classify the degree of chronic inflammation in hematoxylin and eosin-stained adjacent sections of biopsy samples as one of four grades (0, 1+, 2+, or 3+).

We developed a semi-quantitative scoring system as a practical method of determining the frequency of *P*. *acnes*-positive glands in pathology laboratories. In the scoring system, a score of 0 or 1 was assigned to each core obtained by a single prostate biopsy, according to the absence or presence of at least one *P*. *acnes*-positive gland in each core under microscopic observation (x200 magnification). The mean score for each biopsy sample was calculated as the total score of all cores divided by the total number of cores for each biopsy.

The frequency or mean score of *P*. *acnes*-positive glands was basically evaluated without information about the final diagnosis, although observers may have recognized cancer lesions in the last biopsy samples of the PCa patients.

### Statistical analyses

The frequency or mean score of *P*. *acnes*-positive glands, number of *P*. *acnes*-positive macrophages per core, grade of chronic prostatitis of each biopsy sample, and serum PSA titer of the patient just before each biopsy were compared between the first and last biopsy from PCa or control patients using the Wilcoxon signed-rank test, and between PCa and control patients using the Mann-Whitney U test. ROC curves were plotted to evaluate the sensitivity and specificity of the frequency or mean score of *P*. *acnes*-positive glands in biopsy samples, and the PSA titer of patients just before each biopsy in the diagnosis of PCa. GraphPAD PRISM Ver.6 (GraphPAD Software, Inc., San Diego, CA, USA) was used for these analyses. Univariate and multivariate logistic regression analyses were used to examine the risk factors for PCa, where the independent variables were frequency or mean score of *P*. *acnes*-positive glands, number of *P*. *acnes*-positive macrophages per core, grade of chronic inflammation of each biopsy, and serum PSA titer of patients just before each biopsy. The analyses were performed with IBM SPSS statistics ver. 22.0 (IBM Co., Armonk, NY, USA). A p-value of less than 0.05 was considered to be statistically significant.

## Results

### Localization of *P*. *acnes*

**Fig 2:**
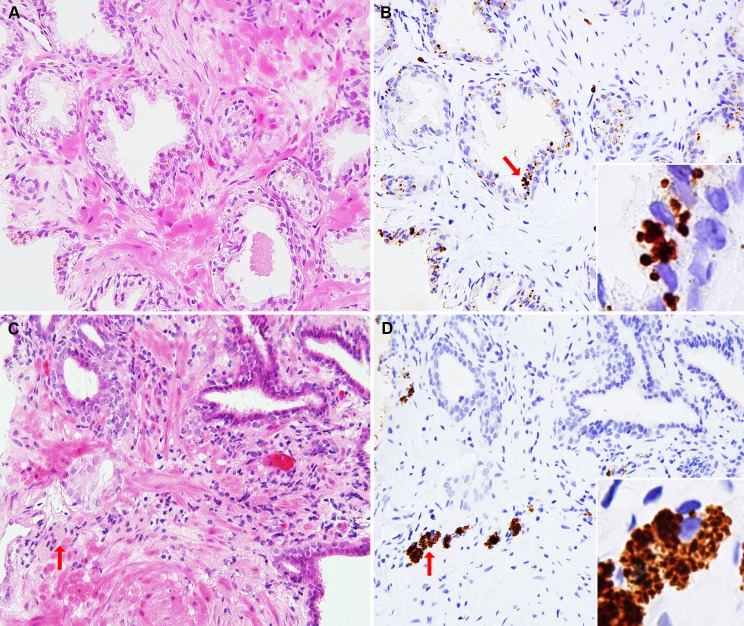
*P*. *acnes*-positive glands and *P*. *acnes*-positive macrophages in the negative prostatic needle biopsy samples detected by IHC with PAL antibody. Two representative areas with many *P*. *acnes*-positive glands or *P*. *acnes*-positive macrophages are shown pairwise (A-B or C-D, respectively) with hematoxylin and eosin staining (A, C) and IHC with the PAL antibody (B, D). A-B: Many *P*. *acnes*-positive glands are clustered in an area of the first negative biopsy sample from a patient with PCa. The antibody reacted with a few small round bodies in some epithelial cells, as shown in the inset, of the gland indicated by an arrow. C-D: *P*. *acnes*-positive macrophages are clustered in some areas of stromal inflammatory cell infiltration of the first negative biopsy samples from a patient with PCa. The antibody reacted with many small round bodies, as shown in the inset, of the macrophages indicated by an arrow.

In all biopsied specimens, the PAL antibody reacted with small round bodies in the epithelial cells of prostatic glands, and we detected PAL antibody-positive small round bodies in macrophages scattered throughout the prostatic stromal areas (Fig 2). No positive signal was observed in the negative-control experiments where IHC was performed without PAL-antibody (S2 Fig). The morphology of the bodies detected with the PAL antibody was similar to that of cultured epithelial cells infected with *P*. *acnes* in vitro at 5 days postinfection and that of prostate glandular epithelial cells in mice infected by transurethral injection of *P*. *acnes* in vivo at 1 or 2 weeks postinfection (S3 and S4 Figs). The number of *P*. *acnes*-positive glands and the number of *P*. *acnes*-positive macrophages differed in each of many cores obtained by identical biopsy.

### Frequency of *P*. *acnes*–positive glands

**Fig 3:**
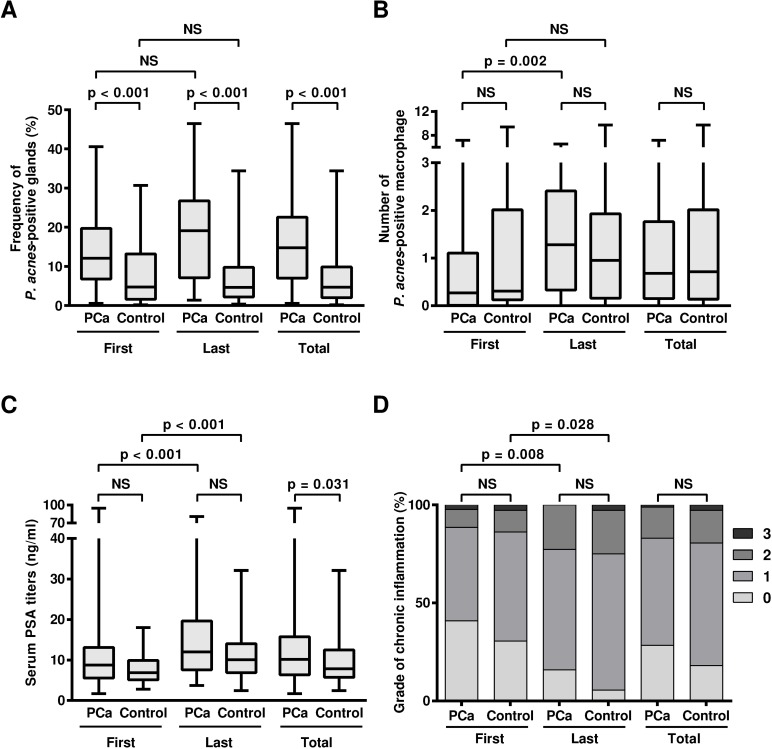
Frequency of *P*. *acnes*-positive glands, number of *P*. *acnes*-positive macrophages, serum PSA titers, and grade of chronic inflammation. A) Frequency (%) of *P*. *acnes*-positive glands. B) Number of *P*. *acnes*-positive macrophage. C) Serum PSA titer. D) Grade of chronic inflammation. PCa: samples from PCa patients, Control: samples from control patients, First: first biopsy samples, Last: last biopsy samples, Total: first and last biopsy samples combined, NS: not significant.

**Fig 4:**
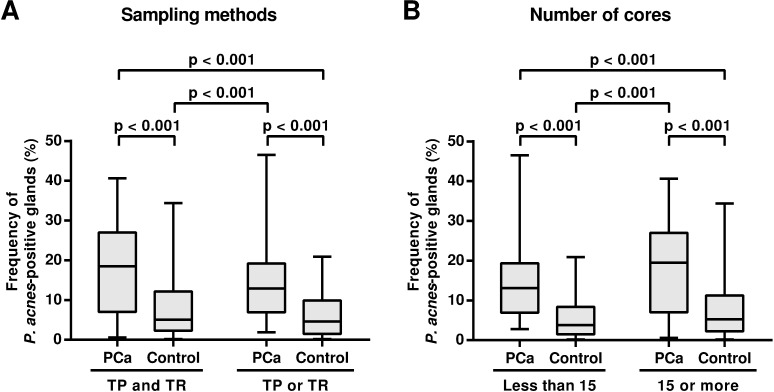
Frequency of *P*. *acnes*-positive glands in samples obtained by different sampling methods or by biopsy with different number of cores. A) Comparison between PCa and control samples obtained using the transperineal (TP) or transrectal (TR) method, and by a combination of these methods (TP and TR). B) Comparison between the PCa and control samples obtained by biopsy with less than 15 cores and by biopsy with 15 or more cores.

In the PCa patients, the median frequency (%) of prostate glands with intraepithelial *P*. *acnes* was 12.1 and 19.1 in the first and last biopsy samples, respectively, and the difference was not significant (Fig 3A). In the control patients, the median frequency was 4.8 and 4.7 in the first and last biopsy samples, respectively, and the difference was not significant. The frequency was significantly higher in the PCa patients than control patients for both the first and last biopsy samples (P’s < 0.001). When the samples from the first and last biopsy were combined, the median frequency was 14.8 in samples from the PCa patients and 4.7 in samples from the control patients (P < 0.001). The increase in the frequency of *P*. *acnes*-positive glands in the PCa samples was also found in the results obtained using a different sampling method (transperineal or transrectal) as well as in the results obtained by biopsy with a different number of cores sampled (≥15 cores or < 15 cores; Fig 4).

### Number of *P*. *acnes*–positive macrophages

The number of stromal macrophages with *P*. *acnes* per core was higher in the last biopsy than the first biopsy samples from the PCa patients (P = 0.002), but the difference was not significant between the first and last biopsy samples from the control patients (Fig 3B). The number of *P*. *acnes*-positive macrophages in the first and last biopsy samples, or when all the samples from the first and the last biopsy were combined, was not different between the PCa and control patients.

### Serum PSA titers just before each biopsy

In the PCa patients, the median titer of serum PSA (ng/ml) was 8.8 and 12.0 at the first and last biopsy, respectively, with significant increase (P < 0.001) detected at the last biopsy (Fig 3C). In control patients, the median titer of the serum PSA was 6.9 and 10.1 at the first and last biopsy, respectively, with a significant increase in PSA titer (P < 0.001) detected at the last biopsy. Although the median serum PSA titer was a little bit higher in the PCa group than in control patients (P = 0.031), the difference was not significant between the PCa and control patients at the first and last biopsy when all values at the first and last biopsy samples were combined.

### Grade of chronic prostatitis

The chronic inflammation grade was higher in the last biopsy samples than in the first biopsy samples for both the PCa and control patients (P = 0.008 and 0.028, respectively; Fig 3D). The degree of chronic inflammation was not different between the PCa and control patients in the first and the last biopsy samples, or when all of the samples from the first and last biopsies were combined.

### ROC curves for detecting PCa

**Fig 5:**
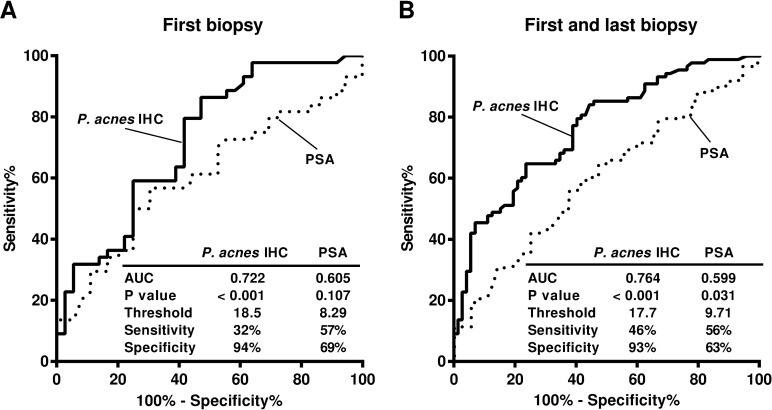
Receiver operating characteristic curves for the frequency of *P*. *acnes*-positive glands and serum PSA titer. A) Analysis of the results from first biopsy samples with and without PCa. B) Analysis of the results from both the first and last biopsy samples with and without PCa. *P*. *acnes* IHC: the frequency of *P*. *acnes*-positive glands, PSA: serum PSA titer

ROC curves in the diagnosis of PCa were made based on the frequency of *P*. *acnes*-positive glands in the first negative biopsy samples or in all samples from the first and last biopsy combined (Fig 5). For comparison, ROC curves for the diagnosis of PCa were also made with the serum PSA titer at the first biopsy and all the values at the first and the last biopsy combined. The AUC was higher with the frequency of *P*. *acnes*-positive glands than the serum PSA titer in each setting (0.722 vs. 0.605 and 0.764 vs. 0.599, respectively). Specificity was higher with the frequency of *P*. *acnes*-positive glands than the serum PSA titer in each setting (94% vs. 69% and 93% vs. 63%, respectively). Sensitivity was lower with the frequency of *P*. *acnes*-positive glands than the serum PSA titers in each setting (32% vs. 57% and 46% vs. 56%, respectively). The threshold frequency of *P*. *acnes*-positive glands was 18.5% and 17.7% in each setting. The threshold value of the serum PSA titer was 8.29 and 9.71 ng/ml in each setting.

### Logistic regression analysis

Logistic regression analyses with the results from the first biopsy samples or the results from the first and last biopsy samples combined, the frequency of *P*. *acnes*-positive glands and a serum PSA titer higher than the threshold obtained by each ROC curve were extracted as independent risk factors (Table 2). The odds ratio for the frequency of *P*. *acnes*-positive glands was 14.8 (P = 0.003) and 13.9 (P < 0.001) by multivariate analysis in each setting, respectively. The odds ratio for the serum PSA titer was 4.6 (P = 0.006) and 2.3 (P = 0.022) by multivariate analysis in each setting, respectively. The number of *P*. *acnes*-positive macrophages per core and the grade of chronic inflammation were not risk factors as determined by univariate or multivariate analysis.

**Table 2:** Logistic regression analysis with the frequency of *P*. *acnes*-positive glands.

	Threshold	Univariate			Multivariate		
		OR	95%CI	P	OR	95%CI	P
First biopsy							
Frequency of *P*. *acnes*-positive glands	18.5	7.9	1.7–37.8	0.009	14.8	2.5–86.4	0.003
Number of *P*. *acnes*-positive macrophage	0.36	0.8	0.4–2.1	NS	0.5	0.2–1.6	NS
Chronic inflammation grade	2	0.8	0.2–3.0	NS	0.3	0.1–2.0	NS
Serum PSA titers	8.29	3.0	1.2–7.6	0.021	4.6	1.5–13.8	0.006
Total of first and last biopsy							
Frequency of *P*. *acnes*-positive glands	17.7	11.2	4.1–30.3	< 0.001	13.9	4.8–40.3	< 0.001
Number of *P*. *acnes*-positive macrophage	0.15	1.5	0.7–3.0	NS	0.9	0.4–2.1	NS
Chronic inflammation grade	2	0.9	0.4–1.9	NS	0.4	0.2–1.2	NS
Serum PSA titers	9.71	2.1	1.1–4.0	0.023	2.3	1.1–4.8	0.022

OR: odds ratio, CI: confidence interval, NS: not significant

### A semi-quantitative scoring system for practical use

**Fig 6:**
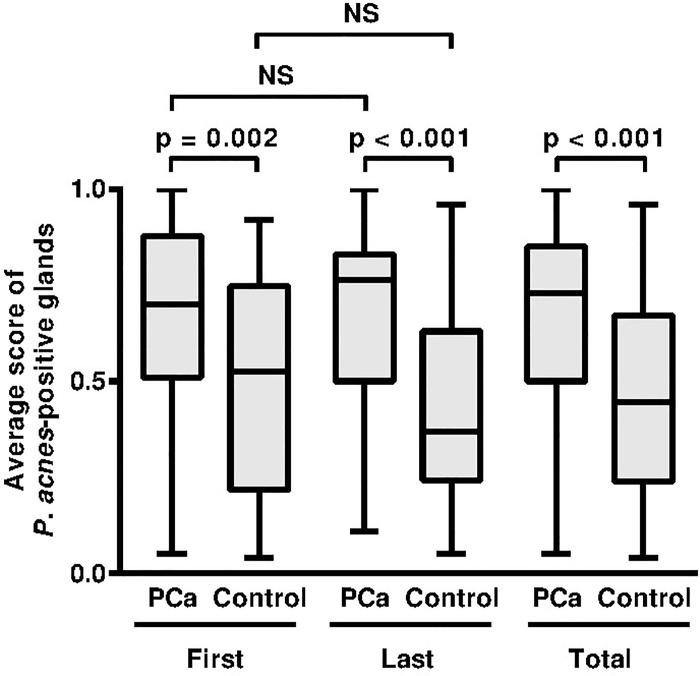
Mean score of *P*. *acnes*-positive glands. PCa: samples from PCa patients, Control: samples from control patients, First: first biopsy samples, Last: last biopsy samples, Total: first and last biopsy samples combined, NS: not significant.

In the PCa patients, the median of the mean score of *P*. *acnes*-positive glands was not significantly different between the first (0.700) and last (0.765) biopsy samples (Fig 6). In control patients, the median mean score was not significantly different between the first (0.525) and last (0.370) biopsy samples. The mean score for the first biopsy samples and last biopsy samples was significantly higher in PCa patients than in control patients (P = 0.002 and P < 0.001, respectively). When all samples from the first and last biopsy were combined, the median of the mean score of the PCa patient samples (0.730) was significantly higher than that of the control patient samples (0.445; P < 0.001).

**Fig 7:**
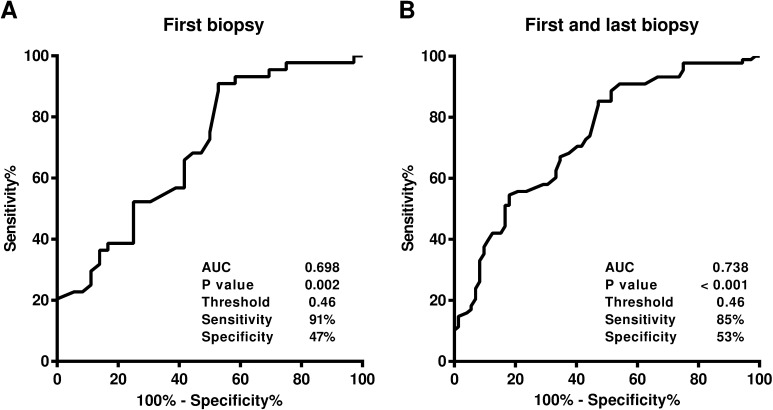
Receiver operating characteristic curves for the mean score of *P*. *acnes*-positive glands. A) Analysis of the results from first biopsy samples with and without PCa. B) Analysis of the results from both the first and last biopsy samples with and without PCa.

ROC curves in the diagnosis of PCa were created with the semi-quantitative scoring system of *P*. *acnes*-positive glands in the first negative biopsy samples or in all samples from the first and last biopsy combined, respectively (Fig 7). The AUC was 0.698 and 0.738 in each setting and these values were a little bit lower than those of the frequency and higher than those of the serum PSA. The sensitivity of the scoring system was 91% and 85% in each setting–far higher than those of both the frequency and serum PSA. The specificity of the scoring system was 47% and 53% in each setting–lower than those of both the frequency and serum PSA. The threshold mean score of *P*. *acnes*-positive glands was 0.46 in both settings.

Logistic regression analysis was performed using the mean score of *P*. *acnes*-positive glands. Univariate analysis revealed that a mean score of 0.46 or more was a significant risk factor with an odds ratio of 8.9 and 6.4 in each setting (P’s < 0.001). Multivariate analysis revealed that a mean score of 0.46 or more was an independent risk factor (Table 3). The odds ratio for the mean score was 14.2 for both settings (P’s < 0.001). The odds ratio for the serum PSA titer was 5.8 and 5.4 in each setting, respectively (P = 0.005 and P < 0.001).

**Table 3:** Logistic regression analysis with the mean score of *P*. *acnes*-positive glands.

	Threshold	Multivariate		
		OR	95%CI	P
First biopsy				
Average score of *P*. *acnes*-positive glands	0.46	14.2	3.5–58.5	< 0.001
Number of *P*. *acnes*-positive macrophage	0.36	0.5	0.2–1.6	NS
Chronic inflammation grade	2	0.7	0.1–3.8	NS
Serum PSA titers	8.29	5.8	1.7–19.9	0.005
Total of first and last biopsy				
Average score of *P*. *acnes*-positive glands	0.46	14.2	5.4–37.8	< 0.001
Number of *P*. *acnes*-positive macrophage	0.15	0.8	0.4–2.0	NS
Chronic inflammation grade	2	0.5	0.2–1.3	NS
Serum PSA titers	9.71	5.4	2.2–13.3	< 0.001

OR: odds ratio, CI: confidence interval, NS: not significant

## Discussion

The frequency of *P*. *acnes*-positive glands was higher in samples from PCa patients than control patients in the prostate core needle biopsy samples, as previously found in radical prostatectomy specimens. The increased frequency of *P*. *acnes*-positive glands in patients with PCa suggests not only that this indigenous bacterium contributes to prostate carcinogenesis but also that the frequency can be used in the risk assessment for patients after the first negative prostate biopsy performed due to an increased PSA titer. According to the results obtained in the present study with limited numbers of PCa and control patients who underwent repeated biopsy in our university hospital, the risk that the first biopsy was a false negative was 14 times higher in patients with than in those without an increased frequency of *P*. *acnes*-positive glands in their biopsy samples, and the risk assessment power with the first negative biopsy samples was superior to the serum PSA titer of patients at the time of the first biopsy.

A limitation of the present study is the selection of the patients with or without PCa. Unlike in our previous study with radical prostatectomy specimens, only prostate biopsy samples from patients who received repeated biopsy in our university hospital were used in the present study. Patients were designated PCa patients when the repeated biopsy finally revealed PCa lesions within 4 years of the first negative biopsy and control patients without PCa when the repeated biopsy did not reveal PCa lesions for at least 3 years (up to 11 years) after the first negative biopsy. In this study setting, most of the PCa patients may have harbored cancer at the time of the first negative biopsy, but the cancer detected in the last biopsy may have occurred during the 4 years after the first negative biopsy. The latter possibility does not refute the implication of prostate *P*. *acnes* infection status in the risk assessment of patients with the first negative biopsy. Alternatively, most of the control patients may not have harbored cancer at the time of the first and the last biopsy, but some of these patients may have harbored cancer that was not detected even by repeated biopsy for at least 3 years (up to 11 years). The latter possibility is thought to be unlikely because identical results for the difference in any parameters between the PCa and control patients were obtained between the first and last biopsy samples. Based on these assumptions, the ROC curves were made and univariate and multivariate logistic regression analyses performed not only with the results from the first biopsy samples but also with the results from all of the samples from the first and last biopsy, to increase the number of samples thereby increasing the power of the analysis.

The lack of a difference in the number of *P*. *acnes*-positive macrophages between the PCa and control samples requires some discussion because significant differences (P = 0.014 in peripheral zone and P = 0.036 in transitional zone) were detected in a previous study with radical prostatectomy specimens [7]. It is most likely that the difference in the results was due to differences in the amount of tissue used in these studies because *P*. *acnes*-positive macrophages are scattered and generally few in number even in a whole prostate tissue section. Indeed, the median number of *P*. *acnes*-positive macrophages was less than two per single core in each group of biopsy samples. Another possibility is that the number of *P*. *acnes*-positive macrophages, which is reported to correlate with the grade of chronic inflammation [7], is not directly associated with prostate carcinogenesis. This possibility is supported by the fact that no difference was detected in the grade of chronic inflammation between the PCa and control patient samples, as shown in the present study and in previous reports [12–14].

Similarly, the higher number of *P*. *acnes*-positive macrophages in the last biopsy samples compared the first biopsy samples from the PCa patients might be reflected in the higher grade of chronic inflammation in the last biopsy sample compared the first biopsy sample from the PCa patients. A higher grade of chronic inflammation in the last than in the first biopsy samples and higher serum PSA titer values at the last biopsy sampling than at the first biopsy sampling were commonly observed in both PCa and control patients and may be partially due to age-related changes or preceding biopsy-related events including inflammation and hyperplasia. A significantly higher increase in the serum PSA titer at the last biopsy in the PCa than in the control patients is presumably caused by cancer progression during the interval between the first and last biopsy.

The sampling method for prostate needle biopsy varies in our university hospital, and may include the transperineal or transrectal method, or a combination of these methods. The number of *P*. *acnes*-positive glands also varies by each core with no remarkable tendency according to the sampling region. Variations in the sampling method and the number of *P*. *acnes*-positive glands per core were corrected by determining the frequency of *P*. *acnes*-positive glands obtained by counting *P*. *acnes*-positive or -negative prostate glands in all cores of each biopsy sample. Although in the present study, we found no significant difference in the results obtained by different sampling methods or in the results obtained by biopsy with different numbers of cores sampled, a constant and adequate number of cores obtained using an identical sampling method would be desirable to more accurately determine and compare the frequency of *P*. *acnes*-positive glands.

Based on the ROC curve analysis in the diagnosis of PCa, the frequency of *P*. *acnes*-positive glands showed a higher AUC and specificity than those of the serum PSA titer, and logistic regression analysis revealed that the frequency of *P*. *acnes*-positive glands is an independent risk factor for PCa. Although the serum PSA titer was also an independent risk factor, the odd’s ratio for the frequency of *P*. *acnes*-positive glands was much higher than that of the serum PSA titer. These results suggested that the frequency of *P*. *acnes*-positive glands can be used independently and more effectively than the serum PSA titer in the risk assessment for patients with negative results of a first prostate biopsy performed due to an increased serum PSA.

Moreover, in the present study, we used a semi-quantitative scoring system that can be easily performed by pathologists to measure the *P*. *acnes* infection status of prostate biopsy samples. In the scoring system, the pathologist only needs to determine if any *P*. *acnes*-positive glands are present for each core and does not need to count all of the prostate glands with or without a *P*. *acnes*-positive signal. The scoring system worked very well in the present study, and the AUC and odds ratios obtained using the scoring system were similar to those obtained by counting all of the glands. Compared to the frequency counting method, the scoring method increased the sensitivity and decreased the specificity for the diagnosis of PCa, making the present scoring method beneficial for practical use because sensitivity is more important than specificity in this type of risk assessment.

Prostate needle biopsy has now become an essential and useful method to make the final diagnosis of PCa, but the large proportion of false negative results is a problem [15]. Moreover, adverse effects caused by needle biopsy, such as erectile dysfunction, is another problem that cannot be ignored [16,17]. The method and technology of needle biopsy is constantly advancing [10,18,19], and the diagnostic accuracy of PCa has been improved by combining tests, such as MRI [19,20], urinalysis [21], and ultrasonography. The development of additional approaches for histologic assessment of biopsy samples provides more information for the final diagnosis.

IHC with the PAL antibody may be able to decrease the number of needle biopsies and avoid adverse effects of surgical stress, thereby reducing the burden on patients with low risk of PCa. We propose the following method for applying the frequency of *P*. *acnes*-positive glands to PCa diagnosis. When the first needle biopsy performed due to an increased PSA titer does not support a diagnosis of PCa, we propose that the mean score of *P*. *acnes*-positive glands be determined using IHC with the PAL antibody. If the mean score of *P*. *acnes*-positive glands is lower than 0.46, the patient would be considered to have a low risk of PCa and would not require close observation. If the mean score is 0.46 or higher, the patient would be considered to have a high risk of PCa and careful follow-up (including re-biopsy) is recommended. Thus, the *P*. *acnes* infection status of the first negative prostate biopsy can be supportive information for urologists in planning repeated biopsy or follow-up strategies.

## Supporting Information

S1 Fig IHC with PAL antibody can detect both phylotype I and II. Cultured macrophages (Raw 264) infected by either phylotype I or II for 2 h were immunostained with PAL antibody. A: a macrophage infected by phylotype I *P*, *acnes*, B: a macrophage infected by phylotype II *P*. *acnes*.
(TIF)

S2 Fig IHC with and without PAL-antibody in the prostate biopsy specimen. A: with PAL-antibody, B: without PAL-antibody.
(TIF)

S3 Fig Coccoid structures of intracellular *P*. *acnes* in human epithelial cells (A549) observed at 5 days postinfection. A-D: 1 day postinfection, E-H: 5 days postinfection, A-C and E-G: with PAL-antibody, D and H: without PAL-antibody.
(TIF)

S4 Fig Coccoid structures of intracellular *P*. *acnes* in prostate glands of mice infected by transurethral injection. A and B: 1 week postinfection, C and D: 2 weeks postinfection, A and C: with PAL-antibody, B and D: without PAL-antibody.
(TIF)
