# Supplementary figures and images for: Frequency of Propionibacterium acnes Infection in Prostate Glands with Negative Biopsy Results Is an Independent Risk Factor for Prostate Cancer in Patients with Increased Serum PSA Titers
Source: PLoS One. 2017 Jan 12;12(1):e0169984. doi: 10.1371/journal.pone.0169984 (PMC5231393; doi:10.1371/journal.pone.0169984)

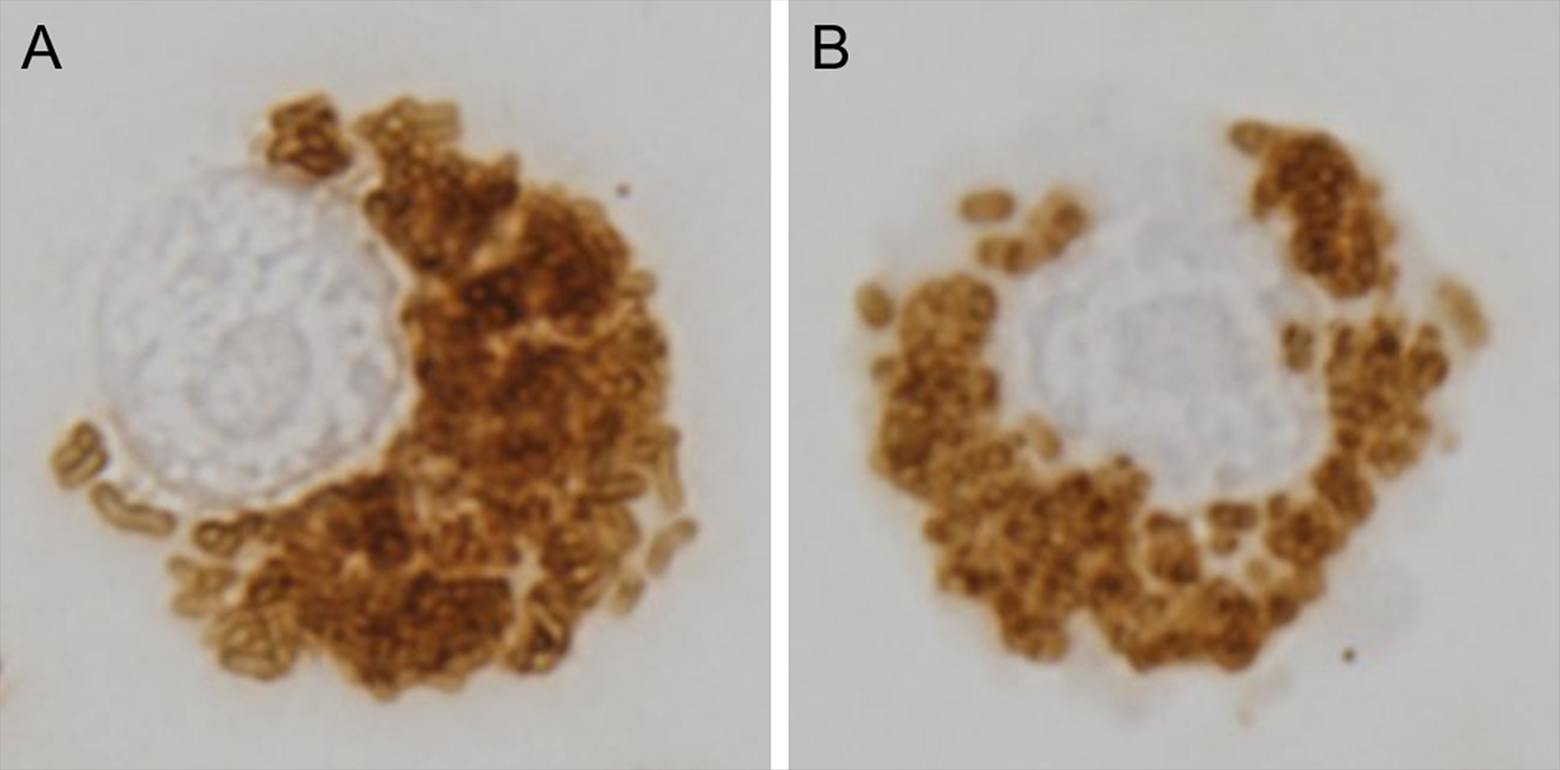

Supplement: S1 Fig — Cultured macrophages (Raw 264) infected by either phylotype I or II for 2 h were immunostained with PAL antibody. A: a macrophage infected by phylotype I P, acnes, B: a macrophage infected by phylotype II P. acnes. (TIF) [file pone.0169984.s001.tif]

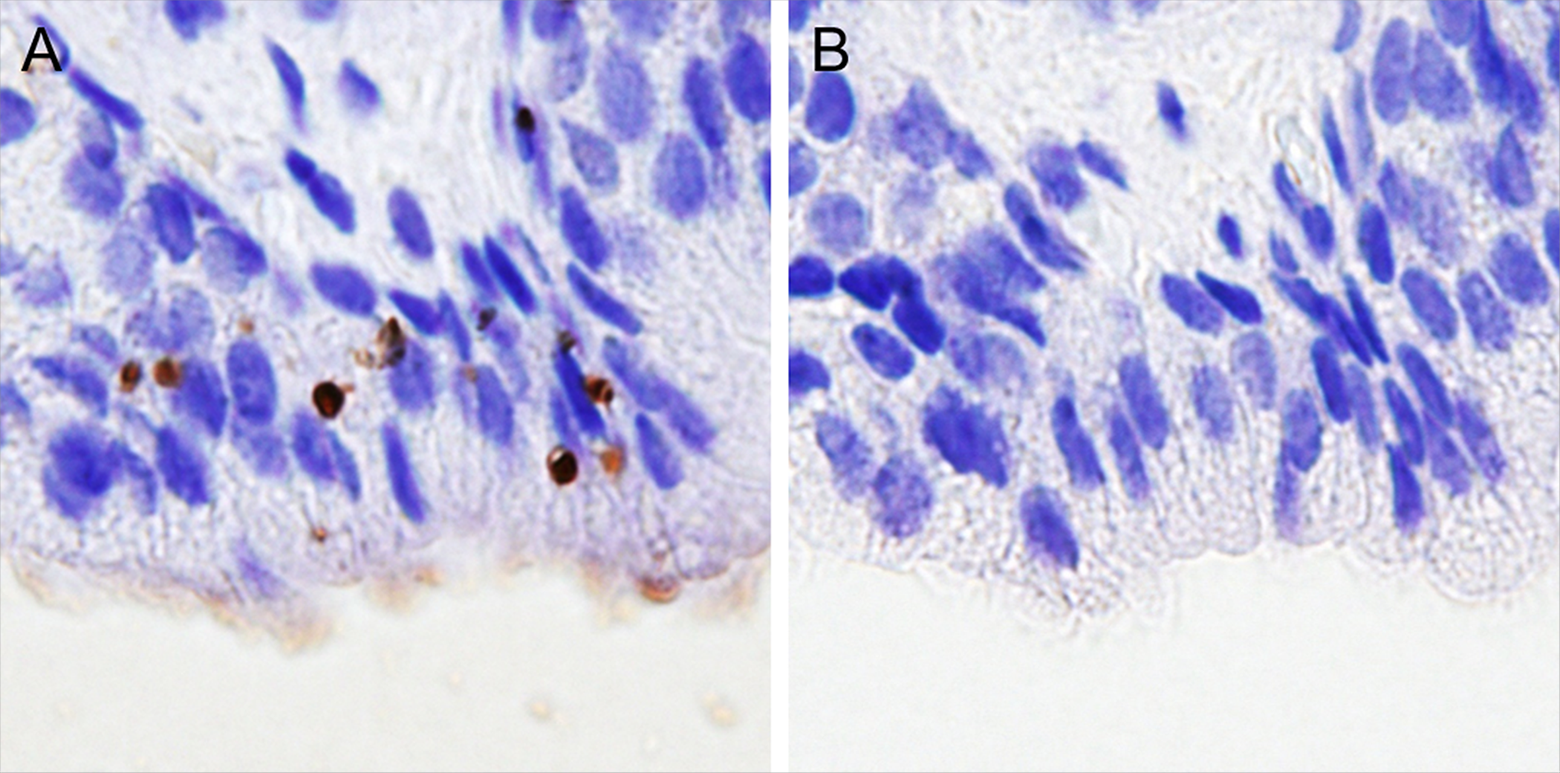

Supplement: S2 Fig — A: with PAL-antibody, B: without PAL-antibody. (TIF) [file pone.0169984.s002.tif]

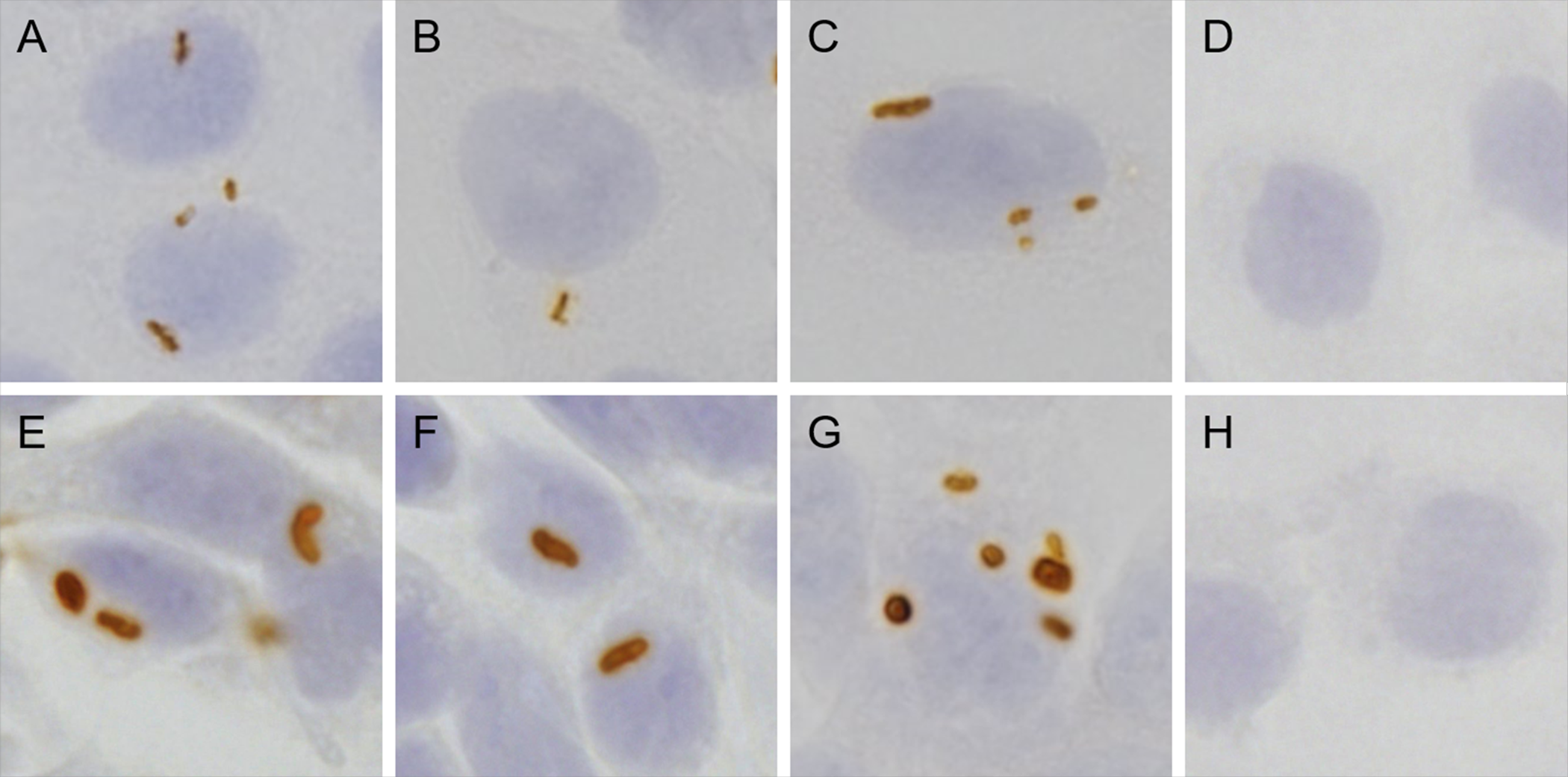

Supplement: S3 Fig — A-D: 1 day postinfection, E-H: 5 days postinfection, A-C and E-G: with PAL-antibody, D and H: without PAL-antibody. (TIF) [file pone.0169984.s003.tif]

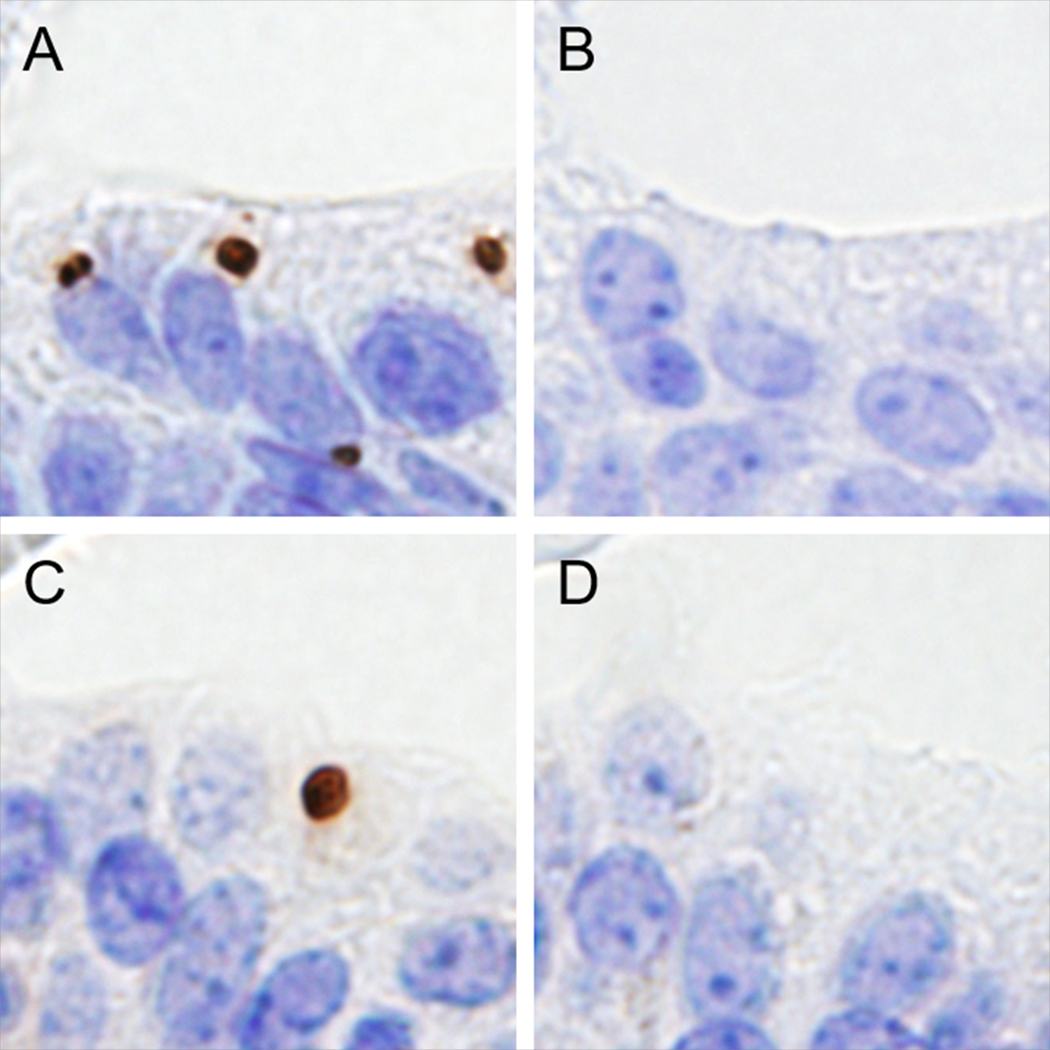

Supplement: S4 Fig — A and B: 1 week postinfection, C and D: 2 weeks postinfection, A and C: with PAL-antibody, B and D: without PAL-antibody. (TIF) [file pone.0169984.s004.tif]
